# Comparison of Several Adiposity Indexes in Predicting Hypertension among Chinese Adults: Data from China Nutrition and Health Surveillance (2015–2017)

**DOI:** 10.3390/nu15092146

**Published:** 2023-04-29

**Authors:** Yuge Li, Dongmei Yu, Yuxiang Yang, Xue Cheng, Wei Piao, Qiya Guo, Xiaoli Xu, Liyun Zhao, Yuying Wang

**Affiliations:** 1 National Institute for Nutrition and Health, Chinese Center for Disease Control and Prevention, Beijing 100050, China; liyuge1122@163.com (Y.L.); yudm@ninh.chinacdc.cn (D.Y.); yxyang_ninhccdc@126.com (Y.Y.); piaowei@ninh.chinacdc.cn (W.P.); guoqy@ninh.chinacdc.cn (Q.G.); xuxl@ninh.chinacdc.cn (X.X.); zhaoly@ninh.chinacdc.cn (L.Z.); 2 NHC Key Laboratory of Trace Element Nutrition, National Institute for Nutrition and Health, Chinese Center for Disease Control and Prevention, Beijing 100050, China

**Keywords:** adiposity index, hypertension, Chinese adult, nutrition surveillance

## Abstract

The current study is to explore the association of the Chinese visceral adiposity index (CVAI) with hypertension, and to compare the predictive power of different adiposity indexes regarding hypertension among Chinese adults aged over 45 years. A total of 99,201 participants aged over 45 years from the China Nutrition and Health Surveillance 2015–2017 were included in this study. Multivariate adjusted logistic regression was used to calculate the odds ratio (OR) and 95% confidence interval (CI) of hypertension. Multivariate adjusted restricted cubic spline analyses were applied to explore the association of adiposity indexes with hypertension. Receiver operating characteristic (ROC) analyses were used to compare the predictive powers of different adiposity indexes of hypertension. All eight adiposity indexes included in this study were positively associated with hypertension. Compared with those in the lowest quartile of the CVAI, the participants in the highest quartile showed a significantly higher risk of hypertension (OR = 3.70, 95% CI = 3.54–3.86) after multiple adjustments. The ROC analyses suggested that the CVAI was the strongest predictor of hypertension compared to other adiposity indexes in both genders. The findings supported that the CVAI could serve as a reliable and cost-effective method for early identifying hypertension risk.

## 1. Introduction

During the last three decades, the number of hypertensive patients aged from 30 to 79 has increased from 650 million in 1990 to 1.28 billion in 2019 globally. Among those hypertensive patients, nearly half did not report having been previously diagnosed with hypertension [1]. In China, hypertension is the primary cause of cardiovascular diseases. The prevalence of hypertension has been increasing rapidly, which has further imposed heavy burdens on the medical care system [2]. Therefore, an earlier diagnosis of hypertension is of great necessity to reduce the risk of adverse health outcomes.

Among a wide spectrum of hypertension-related risk factors, adiposity is less controversial [3]. Previous evidence has indicated that visceral adipose tissue, rather than subcutaneous adipose tissue, was more strongly associated with the risk of hypertension [4,5]. Although visceral adipose tissue can be evaluated using computed tomography (CT) and magnetic resonance imaging (MRI), they are not suitable for large population-based studies because of the high cost and potential exposure risk. Body mass index (BMI) and waist circumference (WC), generally used to reflect adiposity, are incapable of identifying adipose tissue in the abdominal region. Several adiposity indexes developed to facilitate the screening of hypertension have emerged recently, including the visceral adiposity index (VAI), lipid accumulation product index (LAP), body roundness index (BRI), body shape index (ABSI), and conicity index (CI) [6,7,8,9,10]. However, previous studies have indicated that these indexes may have limitations in predicting hypertension among Chinese populations [11,12,13].

The Chinese visceral adiposity index (CVAI) was developed by Xia et al. in 465 Chinese adults to diagnose visceral adiposity, and its predictive power has been validated in a cross-sectional study [14]. Meanwhile, this index has been proven to be associated with some diseases, including metabolic-associated fatty liver disease [15], diabetes [16], diabetic complications [17], carotid plaque [18], and renal damage [19]. Nevertheless, little attention is paid to the connection between hypertension and the CVAI among middle-aged and elderly Chinese people [20]. The accuracy of different adiposity indexes in identifying hypertension remains to be verified.

The current study aims to explore the association of the CVAI with hypertension and to compare the predictive powers of different adiposity indexes (CVAI, VAI, LAP, BRI, ABSI, CI, WC, and BMI) regarding hypertension among Chinese middle-aged and elderly people, based on a nationally representative survey.

## 2. Materials and Methods

### 2.1. Participants

All the data in the study were obtained from the China Nutrition and Health Surveillance (2015–2017) (CNHS 2015–2017). The multistage stratified random sampling method was adopted to obtain a representative national sample in 2015–2017. All the participants were 18 years old and above and were from 31 provinces/municipalities/autonomous regions in mainland China. The details of the design and sampling method of the CNHS are available in the previous publication [21]. Participants without complete blood pressure or anthropometric measurement information were excluded. Finally, a total of 99,201 participants aged over 45 years were included in this study.

The CNHS 2015–2017 was approved by the Ethics Review Committee of the Chinese Center for Disease Control and Prevention (No. 2015-19B, in June 2015), and all the subjects had signed the informed consent before the survey.

### 2.2. Basic Information Collection

Information related to gender, age, geographic region (urban/rural area), educational level, marital status, income, family history, lifestyle factors (smoking and alcohol consumption, sleep duration, physical activity, sedentary behavior, medical examination within one year), and previous disease diagnoses was collected through a standardized questionnaire by well-trained investigators in a face-to-face manner.

### 2.3. Anthropometric Measurement and Definition of Hypertension

To reduce measurement bias, height, weight, waist circumference, and blood pressure were measured by well-trained local CDC staff. Anthropometric measurements were carried out under a fasting condition in the morning in a fixed place. Height was measured with a metal column height meter with an accuracy of 0.1 cm while the participants were standing in an upright position. Weight was measured with an electronic weight scale with an accuracy of 0.1 kg. Waist circumference was measured twice using a tape with an accuracy of 0.1 cm, and the average value was calculated.

Blood pressure was measured three times to the nearest 1 mmHg with the cuff positioned equivalent to the heart level on the left arm by an electronic sphygmomanometer (OMRON HBP-1300) after the participants had rested in a seated position for at least 5 min. The average value of the three-times systolic blood pressure (SBP) and diastolic blood pressure (DBP) was used for the data analysis. Hypertension was defined as SBP ≥ 140 mmHg and (or) DBP ≥ 90 mmHg and (or) receiving antihypertensive medications within two weeks [22].

### 2.4. Laboratory Test

An 8 mL fasting blood sample was collected to measure fasting glucose, total cholesterol, triglyceride (TG), low-density lipoprotein cholesterol (LDL-C), high-density lipoprotein cholesterol (HDL-C), and glycosylated hemoglobin (HbA1c). All the above measurements were taken by professionals following strict quality control standards in the laboratory.

### 2.5. Adiposity Indexes Calculations

The specific formula for calculating the Adiposity Indexes is as follows:

(1) $$\mathrm{BMI}=\frac{{\mathrm{Weight}}_{\left(\mathrm{kg}\right)}}{{{\mathrm{Height}}^{2}}_{\left(m\right)}}$$

(2) $$\mathrm{VAI}\;\;\mathrm{for}\;\;\mathrm{males}=\frac{{WC}_{\left(cm\right)}}{39.68+\left(1.88\times BMI\right)}\times \frac{{TG}_{\left(mmol/L\right)}}{1.03}\times \frac{1.31}{{HDL-C}_{\left(mmol/L\right)}}\;\mathrm{VAI}\;\;\mathrm{for}\;\;\mathrm{females}=\frac{{WC}_{\left(cm\right)}}{36.58+(1.89\times BMI)}\times \frac{{TG}_{\left(mmol/L\right)}}{0.813}\times \frac{1.52}{{HDL-C}_{\left(mmol/L\right)}}$$

(3) CVAI  for  males=−267.93+0.68×age+0.03×BMI+4×WCm+22×Log10TG(mmol/L)−16.32×HDL−C(mmol/L)CVAI  for  females=−187.32+1.71×age+4.32×BMI+1.12×WCm+39.76×Log10TG(mmol/L)−11.66×HDL−C(mmol/L)

(4) $$\mathrm{LAP}\;\;\mathrm{for}\;\;\mathrm{males}=\left({WC}_{\left(cm\right)}-58\right)\times {TG}_{\left(mmol/L\right)}\;\mathrm{LAP}\;\;\mathrm{for}\;\;\mathrm{females}=\left({WC}_{\left(cm\right)}-65\right)\times {TG}_{\left(mmol/L\right)}$$

(5) $$\begin{array}{c} ABSI=\frac{{WC}_{\left(m\right)}}{{BMI}^{2/3}\times {{\mathrm{Height}}^{1/2}}_{\left(m\right)}} \end{array}$$

(6) $$\mathrm{BRI}=364.2-365.5\times \sqrt{1-{（\frac{{WC}_{\left(m\right)}}{2\pi }）}^{2}÷{\left(0.5\times {\mathrm{Height}}_{\left(m\right)}\right)}^{2}}$$

(7) $$\mathrm{CI}=\frac{{WC}_{\left(m\right)}}{0.109\times \sqrt{\frac{{\mathrm{Weight}}_{\left(kg\right)}}{{\mathrm{Height}}_{\left(m\right)}}}}$$

where BMI is the body mass index; VAI is the visceral adiposity index; WC is the waist circumference; TG is triglyceride; HDL-C is high-density lipoprotein cholesterol; CVAI is the Chinese visceral adiposity index; LAP is the lipid accumulation product index; ABSI is a body shape index; BRI is the body roundness index; and CI is the conicity index.

### 2.6. Covariates

The covariates in the sample description and logistic regression analysis were as follows. (1) The geographic region was categorized as an urban area or rural area. (2) The education level was categorized as primary school and below, junior middle school, or senior high school and above. (3) Marital status was divided into married or other. (4) Income levels were divided into low (<10,000 CNY), medium (10,000–25,000 CNY), or high (>25,000 CNY), based on the annual household income per capita. (5) According to the WC, the participants were grouped as normal weight or central obesity [23]. (6) According to the BMI, the participants were divided into underweight (BMI < 18.5), normal weight (18.5 ≤ BMI < 24), overweight (24 ≤ BMI < 28), or obese (BMI ≥ 28) [23]. (7) A family history of hypertension (Yes/No) was defined as any one of the lineal relatives (including grandparents, parents, or siblings) who had been diagnosed with hypertension. (8) Smoking status was separated into current smoker or non-smoker. (9) Alcohol drinking was divided into alcohol intake within one month or not. (10) Sleep duration was categorized as <7 h, 7~8 h, or ≥9 h per day. (11) Physical activity was divided into low (MET < 600), medium (600 ≤ MET ≤ 3000), or high (MET > 3000), according to the weekly total metabolic equivalent (MET-min/week) and total duration of different physical activities [24]. (12) Sedentary behavior was grouped as <2 h, 2~3 h, or ≥4 h per day. (13) A medical examination within one year was categorized as yes or no.

### 2.7. Statistical Analysis

Given that the abnormal distribution of our data, the continuous variables in this study are shown as the median with an IQR (interquartile range) and were compared by Wilcoxon tests between the different groups. The categorical variables were described by count (n, %) and compared by chi-square tests between the different groups.

Three multivariate logistic regression models were built to identify the connections between the adiposity indexes and hypertension. The results are presented as the odds ratio (OR) and 95% confidence interval (95% CI). Each adiposity index was divided into quartiles, with the lowest quartile (Q1) as the reference group. Meanwhile, each adiposity index as a continuous variable was examined per SD increment. Model 1 was an unadjusted model. Model 2 was adjusted for age and gender. Model 3 was further adjusted for the geographic region, education, marital status, income, family history, smoking, alcohol drinking, sleeping time, physical activity, sedentary behavior, medical examination within one year, diabetes, and dyslipidemia.

To further analyze the dose–response relationships between hypertension and the adiposity indexes, multivariate-adjusted restricted cubic spline (RCS) analyses were used, and the four knots were set at the 5th, 35th, 65th, and 95th percentiles, with the 10th percentile as the reference knot [25]. Furthermore, receiver operating characteristic (ROC) analyses were used to determine the area under the ROC curves (AUC) between hypertension and the adiposity indexes to compare the predictive powers of the different adiposity indexes of hypertension in males and females. The differences between the different adiposity indexes for hypertension in both genders were determined according to DeLong et al.’s non-parametric approach [26]. The definition of statistical significance was two-sided, *p* < 0.05.

In the current study, SAS version 9.4 software (SAS Institute, Inc., Cary, NC, USA) was used for all the statistical data cleaning. R software version 4.2.2 was used to perform the ROC curve and dose–response analyses.

## 3. Results

### 3.1. Basic Characteristics

The basic characteristics, anthropometric measurements, and laboratory test data of the participants aged 45 years and above in CNHS 2015–2017 are shown in Table 1 and Table 2. As Table 1 shows, 52% of the total participants had hypertension. Of the total, 51.9% of males and 52% of females had hypertension. As Table 2 shows, the average systolic and diastolic blood pressures of patients with hypertension were 152.0 mmHg and 86.33 mmHg, respectively. The hypertensive participants tended to be older and had greater weight, FPG, TC, TG, LDL-C, HbA1c, and lower HDL-C concentrations. Meanwhile, the participants with the following characteristics were more likely to develop hypertension: lower education and income, married, family history of hypertension, central obesity or general obesity, smoking at that time, inadequate sleep duration, longer sedentary behaviors, receiving medical examination within one year, diabetes, and dyslipidemia.

As the results show in Table 3, the hypertensive participants had higher adiposity indexes compared with the non-hypertensive participants (*p* < 0.0001).

### 3.2. Association of Adiposity Indexes and Hypertension Risk

Table 4 shows the associations between these adiposity indexes and hypertension by quartile and per standard deviation (SD) increase, respectively. In all three models, the ORs for hypertension increased significantly with the increasing quartile number for all the adiposity indexes (*p* for trend < 0.0001), suggesting that those in the highest quartile had a significantly higher risk of hypertension compared with participants in the lowest quartile of the adiposity indexes. With Q1 of the CVAI as the reference, the ORs (95% CIs) for hypertension in quartiles 2, 3, and 4, were 1.55 (1.50, 1.61), 2.30 (2.21, 2.39), and 3.70 (3.54, 3.86) in Model 3. Moreover, the risk of hypertension was increased per SD increase in the CVAI. The adjusted OR (95% CI) was 1.68 (1.66, 1.71) in Model 3.

**Figure 1:**
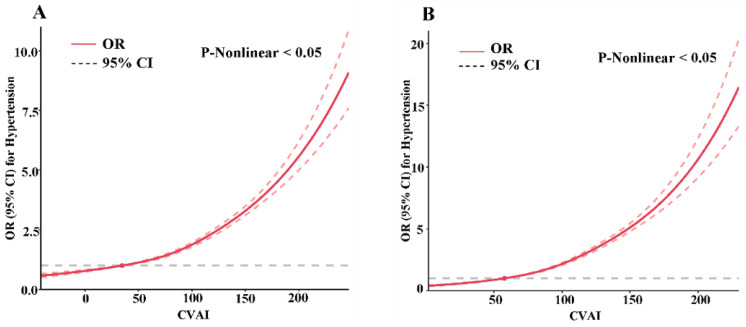
Dose–response relationships between CVAI and risk of hypertension by restricted cubic spline in Chinese 45-years and older males (**A**) and females (**B**). The associations were adjusted for age, geographic region, education, marital status, income, family history, smoking, alcohol drinking, sleeping time, physical activity, sedentary behavior, medical examination within one year, diabetes, and dyslipidemia. The red solid lines and red dashed lines represent the estimated ORs and their 95% CIs respectively. The gray dashed lines represent the corresponding CVAI when the OR is 1.

The dose–response relationship between the ORs (95% CIs) of hypertension and the CVAI is shown in Figure 1. Further information on RCS and the other adiposity indexes and hypertension is available in Supplemental Figure S1. After an adjustment for several confounding factors, a nonlinear association (*p* for nonlinearity < 0.05) was detected between the risk of hypertension and these indexes for both men and women, except for the WC of males (*p* for nonlinearity = 0.058). At the initial increase stage of the CVAI score, the change rate of the OR tended to slightly and then rapidly increase.

### 3.3. Comparison of the Association of CVAI, VAI, LAP, ABSI, BRI, CI, WC, and BMI with Hypertension Risk

**Figure 2:**
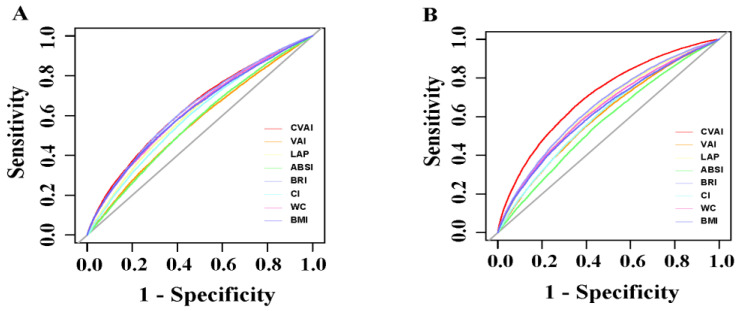
The ROC analysis of adiposity indexes in predicting hypertension in Chinese 45 years and older males (**A**) and females (**B**).

The ROC curves for hypertension and the adiposity indexes for males and females are shown in Figure 2. The ROC curves of all the indexes are above the reference line, indicating that these indexes could be used to identify hypertension. However, among them, the predictive power for hypertension of the CVAI showed to be best for both genders.

Table 5 displays the area under the ROC curves (AUCs) and their 95% CIs for each adiposity index. The AUCs of all the adiposity indexes predicting hypertension were greater than 0.5, and the CVAI (Males: 0.636, 95% CI: 0.631–0.641; Females: 0.706, 95% CI: 0.702–0.710) had the largest AUC among the eight adiposity indexes of hypertension. The differences between the AUC of the CVAI and that of the other indexes for hypertension in both men and women were all significant (*p* < 0.01).

In men, the cutoff with the highest Youden index of the CVAI was 98.268, with a sensitivity of 56.2% and a specificity of 63.80%. In women, the cutoff with the highest Youden index of the CVAI was 101.165, with a sensitivity of 65.31% and a specificity of 65.12%.

## 4. Discussion

This national representative cross-sectional study, containing data on nearly 100,000 individuals, showed that the risk of hypertension in Q4 of the CVAI is more than doubled compared with Q1 of the CVAI after adjusting for potential confounding factors. We also found that the AUC of the CVAI for the diagnosis of hypertension was the largest, and the OR per 1 SD increase in the CVAI with hypertension was the highest when adjusting for the same confounders among all eight adiposity indexes in both genders. These results indicated that the CVAI might have the strongest predictive power to identify hypertension in Chinese middle-aged and older people compared with the VAI, LAP, ABSI, BRI, CI, WC, and BMI.

The CVAI, composed of age, physical measurement data (BMI and WC), and biochemical blood indexes (TG and HDL-C levels), was initially used to reflect the amount of visceral fat and has been established as a reliable indicator for evaluating metabolic health among the Chinese population [14]. Previous studies reported that the CVAI or the VAI was superior to the BMI, WC, ABSI, BRI, CI, or LAP in the diagnosis of hypertension [20,27,28], which is consistent with the results of our study. The reason might be that the BMI alone is not enough to differentiate body fat from lean tissues, and it does not differentiate the location of central fat from peripheral fat [29]. WC and LAP can better reflect abdominal obesity than the BMI but have limitations in distinguishing subcutaneous from visceral adipose tissue [14,30]. The ABSI also has also similar limitations to the BMI and WC [8] and was reported to be a weak index for metabolic syndrome and its components in a few cross-sectional studies [31]. Fujita et al. proved that, compared with BMI or WC, the ABSI was not a better predictor of hypertension in Japanese adults [32]. The possible reason for this may be racial diversity. As widely recommended adiposity indexes, WC and BMI are also alternative predictors for hypertension if the data for blood lipid levels are not available. CI, developed by Valdez et al. in 1991, is calculated by weight, height, and WC [9]. Though there is plenty of evidence indicating that CI is a reliable index for predicting hypertension, in our study it is inferior to the CVAI in identifying hypertension [33,34]. These discrepancies might have several explanations, including ethnic differences, and different sizes and characteristics of the study samples. However, the CVAI and VAI can reflect visceral fat mass [14].

It is noteworthy that the BRI, a novel predictor of visceral adipose tissue and body fat percentage, developed by D.M. Thomas et al. in 2013, also outperformed other adiposity indexes, except the CVAI, as shown in the ROC curves (Figure 2) [7]. Many studies have shown its relationship with hypertension [35,36,37]. However, few studies have compared the predictive ability of the CVAI and the BRI for hypertension simultaneously. Given the excellent performance of the BRI in the current study and the simplicity of its formula, there is a need for further prospective studies to confirm its relationship with hypertension.

It is reported that fat distribution varies by different ethnicities, and Asians could be more prone to have visceral adipose accumulation compared with Caucasians [38,39]. This also reconfirms that the CVAI is more suitable than the VAI for the Chinese population, which is consistent with this study.

Though the positive association between the CVAI and the risk of hypertension has been demonstrated in different populations, there are still considerable studies that reached different conclusions. A cross-sectional study reports that, in adult Chinese, blood pressure is more strongly associated with general adiposity rather than with central adiposity [40]. Our study supports that abdominal obesity is more closely associated with hypertension rather than with generalized obesity. A previous study by Lee et al. supports our results [41]. Han et al., in their prospective cohort study of 10,304 Chinese adults, used the CVAI as a predictor of hypertension and followed up for 6.03 years. Their results indicated that the CVAI could be a reliable index of hypertension in rural Chinese adults [20].

Our study also presents optimal cut-off points for these adiposity indexes. Because it is easy to measure the WC and BMI, the two indexes are commonly used in daily practice to evaluate obesity. The optimal cut-off point for BMI is basically identical to recommendations from the 2018 Chinese Guidelines for Prevention and Treatment of Hypertension [42]. However, the present results show that the optimal cut-off point for WC is inconsistent with the cut-off point according to the guidelines (85 cm for males and 80 cm for females) [42]. Therefore, the WC cut-off points should be reconsidered regarding early detection among Chinese adults with a high risk of hypertension.

Meanwhile, the gender differences in the associations of the CVAI, VAI, LAP, ABSI, BRI, CI, WC, and BMI with hypertension were observed. The AUC values of all the adiposity indexes of the women were higher than those of the men after an adjustment for the same potential confounding factors. Mounting evidence indicates that the predictive powers of adiposity indexes vary by sex [43,44,45]. This could be partly explained by different fat distribution in each gender. It has been discovered that women in Asia tend to carry more fat around their waist than men do, and this may cause more adverse metabolic outcomes in women than men [46]. However, Chen et al. reported that visceral adipose tissue is more prevalent in men rather than women and contributes more to SBP in men rather than in women [44]. Meanwhile, sex hormones and the pathogenesis of hypertension may also cause the differences [47]. Further study needs to be conducted concerning the effects of those potential confounding factors on hypertension. Women may require female-specific and primary prevention and lifestyle management for adiposity and hypertension compared to men.

To the best of our knowledge, this is the first study to compare the CVAI with other obesity indicators, including the VAI, LAP, ABSI, BRI, CI, WC, and BMI, with the risk of hypertension among Chinese people aged 45 years or older recruited by a nationally representative sample. This current study shows the CVAI should be recommended as the best predictor of hypertension in males and females. Furthermore, in the present paper, confounding factors are better controlled.

However, several limitations should be noted in our study. Firstly, the causal relationship between adiposity indexes and hypertension cannot be inferred due to the cross-sectional design. Secondly, although a wide range of confounders were adjusted, residual confounding factors are not taken into consideration, such as dietary factors. Thirdly, as our study only focuses on middle-aged and older Chinese adults, the current results might not be applicable to populations from other age groups.

## 5. Conclusions

In conclusion, all eight adiposity indexes in this study can be used to predict hypertension. The CVAI showed superior predictive powers to the other indexes in middle-aged and elderly Chinese people. The findings demonstrate that the CVAI could serve as a reliable and cost-effective method for the early identification of a high risk of hypertension. It also makes the primary screening and prevention of hypertension among the Chinese population more convenient, thereby reducing the risk of hypertension. Further prospective studies are necessary to validate the connection between the CVAI and adverse health outcomes in other age groups.

## Figures and Tables

**Table 1:** Sample size and basic characteristics of participants aged 45 years and above in CNHS 2015–2017.

Characteristics	Overall			Male			Female		
	Total (*n* = 99,201)	Hypertension (*n* = 51,556)	No Hypertension (*n* = 47,645)	Total (*n* = 47,044)	Hypertension (*n* = 24,428)	No Hypertension (*n* = 22,616)	Total (*n* = 52,157)	Hypertension (*n* = 27,128)	No Hypertension (*n* = 25,029)
Age (years)	59.01 (14.27)	61.62 (14.39)	55.69 (12.84)	59.67 (14.59)	61.89 (14.68)	56.91 (13.34)	58.48 (13.89)	61.39 (14.09)	54.53 (12.33)
Geographic region (n, %)									
Urban	41,848 (42.19)	21,816 (42.32)	20,032 (42.04)	19,217 (40.85)	10,282 (42.09)	8935 (39.51)	22,631 (43.39)	11,534 (42.52)	11,097 (44.34)
Rural	57,353 (57.81)	29,740 (57.68)	27,613 (57.96)	27,827 (59.15)	14,146 (57.91)	13,681 (60.49)	29,526 (56.61)	15,594 (57.48)	13,932 (55.66)
Education level (n, %)									
Primary school or below	55,688 (56.14)	30,398 (58.96)	25,290 (53.08)	22,052 (46.88)	11,711 (47.94)	10,341 (45.72)	33,636 (64.49)	18,687 (68.88)	14,949 (59.73)
Junior middle school	27,458 (27.68)	13,474 (26.13)	13,984 (29.35)	15,768 (33.52)	8013 (32.8)	7755 (34.29)	11,690 (22.41)	5461 (20.13)	6229 (24.89)
Senior high school and above	16,055 (16.18)	7684 (14.90)	8371 (17.57)	9224 (19.61)	4704 (19.26)	4520 (19.99)	6831 (13.1)	2980 (10.98)	3851 (15.39)
Marital status (n, %)									
Married	91,793 (92.53)	46,895 (90.96)	44,898 (94.23)	44,541 (94.68)	22,923 (93.84)	21,618 (95.59)	47,252 (90.6)	23,972 (88.37)	23,280 (93.01)
Other status	7408 (7.47)	4661 (9.04)	2747 (5.77)	2503 (5.32)	1505 (6.16)	998 (4.41)	4905 (9.4)	3156 (11.63)	1749 (6.99)
Income (CNY)									
low	36,316(36.61)	19,729(38.27)	16,587(34.81)	17,585(37.38)	9322(38.16)	8263(36.54)	18,731(35.91)	10,407 (38.36)	8324 (33.26)
medium	40,033(40.36)	20,309(39.39)	19,724(41.4)	18,900(40.18)	9533(39.02)	9367(41.42)	21,133(40.52)	10,776 (39.72)	10,357 (41.38)
high	22,852(23.04)	11,518(22.34)	11,334(23.79)	10,559(22.44)	5573(22.81)	4986(22.05)	12,293(23.57)	5945 (21.91)	6348 (25.36)
Family history									
No	67,948(68.5)	32,703(63.43)	35,245(73.97)	32,860(69.85)	15,809(64.72)	17,051(75.39)	35,088(67.27)	16,894 (62.28)	18,194 (72.69)
Yes	31,253(31.5)	18,853(36.57)	12,400(26.03)	14,184(30.15)	8619(35.28)	5565(24.61)	17,069(32.73)	10,234 (37.72)	6835 (27.31)
WC-based (n, %)									
Normal weight	52,644 (53.07)	23,085 (44.78)	29,559 (62.04)	32,373 (68.81)	14,941 (61.16)	17,432 (77.08)	20,271 (38.87)	8144 (30.02)	12,127 (48.45)
Central obesity	46,557 (46.93)	28,471 (55.22)	18,086 (37.96)	14,671 (31.19)	9487 (38.84)	5184 (22.92)	31,886 (61.13)	18,984 (69.98)	12,902 (51.55)
BMI-based (n, %)									
Underweight	3177 (3.2)	1203 (2.33)	1974 (4.14)	1544 (3.28)	583 (2.39)	961 (4.25)	1633 (3.13)	620 (2.29)	1013 (4.05)
Normal weight	44,134 (44.49)	18,949 (36.75)	25,18 (52.86)	22,209 (47.21)	9643 (39.48)	12,566 (55.56)	21,925 (42.04)	9306 (34.3)	12,619 (50.42)
Overweight	37,074 (37.37)	20,979 (40.69)	16,095 (33.78)	17,331 (36.84)	9970 (40.81)	7361 (32.55)	19,743 (37.85)	11,009 (40.58)	8734 (34.9)
Obesity	14,816 (14.94)	10,425 (20.22)	4391 (9.22)	5960 (12.67)	4232 (17.32)	1728 (7.64)	8856 (16.98)	6193 (22.83)	2663 (10.64)
Current smoker (n, %)									
No	72,691 (73.28)	38,666 (75.00)	34,025 (71.41)	22,479 (47.78)	12,544 (51.35)	9935 (43.93)	50,212 (96.27)	26,122 (96.29)	24,090 (96.25)
Yes	26,510 (26.72)	12,890 (25.00)	13,620 (28.59)	24,565 (52.22)	11,884 (48.65)	12,681 (56.07)	1945 (3.73)	1006 (3.71)	939 (3.75)
Alcohol drinking (n, %)									
NO	72,146 (72.73)	37,485 (72.71)	34,661 (72.75)	25,077 (53.31)	12,763 (52.25)	12,314 (54.45)	47,069 (90.24)	24,722 (91.13)	22,347 (89.28)
YES	27,055 (27.27)	14,071 (27.29)	12,984 (27.25)	21,967 (46.69)	11,665 (47.75)	10,302 (45.55)	5088 (9.76)	2406 (8.87)	2682 (10.72)
Sleep duration (n, %)									
<7 h	25,727 (25.93)	13,894 (26.95)	11,833 (24.84)	11,430 (24.3)	6091 (24.93)	5339 (23.61)	14,297 (27.41)	7803 (28.76)	6494 (25.95)
7~9 h	52,905 (53.33)	26,311 (51.03)	26,594 (55.82)	25,796 (54.83)	12,940 (52.97)	12,856 (56.84)	27,109 (51.98)	13,371 (49.29)	13,738 (54.89)
>9 h	20,569 (20.73)	11,351 (22.02)	9218 (19.35)	9818 (20.87)	5397 (22.09)	4421 (19.55)	10,751 (20.61)	5954 (21.95)	4797 (19.17)
Physical activity (n, %)									
Low	22,033 (22.21)	12,049 (23.37)	9984 (20.95)	11,852 (25.19)	6345 (25.97)	5507 (24.35)	10,181 (19.52)	5704 (21.03)	4477 (17.89)
Moderate	24,859 (25.06)	13,414 (26.02)	11,445 (24.02)	11,436 (24.31)	6299 (25.79)	5137 (22.71)	13,423 (25.74)	7115 (26.23)	6308 (25.2)
High	52,309 (52.73)	26,093 (50.61)	26,216 (55.02)	23,756 (50.5)	11,784 (48.24)	11,972 (52.94)	28,553 (54.74)	14,309 (52.75)	14,244 (56.91)
Sedentary behavior									
0 ~< 2 h	13,295 (13.4)	6716 (13.03)	65,79 (13.81)	5753 (12.23)	2907 (11.9)	2846 (12.58)	7542 (14.46)	3809 (14.04)	3733 (14.91)
2~3 h	38,539 (38.85)	19,519 (37.86)	19,020 (39.92)	18,312 (38.93)	9238 (37.82)	9074 (40.12)	20,227 (38.78)	10,281 (37.9)	9946 (39.74)
≥4 h	47,367 (47.75)	25,321 (49.11)	22,046 (46.27)	22,979 (48.85)	12,283 (50.28)	10,696 (47.29)	24,388 (46.76)	13,038 (48.06)	11,350 (45.35)
Medical examination within one year (n, %)									
NO	70,492 (71.06)	34,900 (67.69)	35,592 (74.70)	19,217 (40.85)	16,719 (68.44)	17,229 (76.18)	36,544 (70.07)	18,181 (67.02)	18,363 (73.37)
YES	28,709 (28.94)	16,656 (32.31)	12,053 (25.30)	27,827(59.15)	7709 (31.56)	5387 (23.82)	15,613 (29.93)	8947 (32.98)	6666 (26.63)
Diabetes (n, %)									
NO	87,241 (87.94)	43,370 (84.12)	43,871 (92.08)	41,494 (88.2)	20,739 (84.9)	20,755 (91.77)	45,747 (87.71)	22,631 (83.42)	23,116 (92.36)
YES	11,960 (12.06)	8186 (15.88)	3774 (7.92)	5550 (11.8)	3689 (15.1)	1861 (8.23)	6410 (12.29)	4497 (16.58)	1913 (7.64)
Dyslipidemia (n, %)									
NO	57,637 (58.10)	27,322 (52.99)	30,315 (63.63)	26,906 (57.19)	13,018 (53.29)	13,888 (61.41)	30,731 (58.92)	14,304 (52.73)	16,427 (65.63)
YES	41,564 (41.90)	24,234 (47.01)	17,330 (36.37)	20,138 (42.81)	11,410 (46.71)	8728 (38.59)	21,426 (41.08)	12,824 (47.27)	8602 (34.37)

Values of polytomous variables may not sum to 100% because of rounding. Abbreviation: WC—waist circumference, BMI—body mass index.

**Table 2:** Anthropometric measurement and laboratory test data of participants aged 45 years and above in CNHS 2015–2017.

Characteristics	Overall				Male				Female			
	Total	Hypertension	No Hypertension	*p*-Value	Total	Hypertension	No Hypertension	*p*-Value	Total	Hypertension	No Hypertension	*p*-Value
Height (cm)	159.00 (12.00)	158.90 (12.00)	159.50 (11.60)	<0.0001	165.00 (9.00)	165.00 (9.00)	165.20 (9.00)	<0.0001	154.20 (8.30)	154.00 (8.10)	155.00 (8.30)	<0.0001
Weight (kg)	61.30 (15.10)	63.00 (15.90)	59.70 (14.00)	<0.0001	65.10 (15.30)	67.10 (16.00)	63.30 (14.20)	<0.0001	58.10 (13.30)	59.80 (14.00)	56.70 (12.30)	<0.0001
SBP (mmHg)	137.33 (27.67)	152.00 (21.34)	125.33 (14.33)	<0.0001	137.00 (26.34)	151.00 (20.00)	125.67 (14.00)	<0.0001	137.33 (29.00)	153.00 (22.33)	125.00 (15.00)	<0.0001
DBP (mmHg)	80.00 (14.67)	86.33 (14.66)	75.00 (11.00)	<0.0001	81.67 (14.66)	88.33 (14.33)	76.67 (10.67)	<0.0001	78.67 (14.67)	84.33 (14.34)	73.67 (11.66)	<0.0001
FPG (mmol/L)	5.29 (0.92)	5.40 (1.02)	5.18 (0.82)	<0.0001	5.30 (0.95)	5.42 (1.03)	5.20 (0.85)	<0.0001	5.28 (0.90)	5.39 (1.01)	5.17 (0.78)	<0.0001
TC (mmol/L)	4.85 (1.23)	4.93 (1.26)	4.78 (1.19)	<0.0001	4.72 (1.20)	4.78 (1.23)	4.66 (1.17)	<0.0001	4.97 (1.24)	5.06 (1.26)	4.89 (1.21)	<0.0001
TG (mmol/L)	1.26 (1.00)	1.37 (1.09)	1.15 (0.88)	<0.0001	1.21 (1.01)	1.29 (1.10)	1.12 (0.91)	<0.0001	1.31 (0.98)	1.44 (1.07)	1.18 (0.85)	<0.0001
LDL-C (mmol/L)	3.02 (1.09)	3.09 (1.12)	2.94 (1.06)	<0.0001	2.92 (1.07)	2.97 (1.08)	2.87 (1.04)	<0.0001	3.11 (1.10)	3.20 (1.13)	3.01 (1.06)	<0.0001
HDL-C (mmol/L)	1.26 (0.44)	1.25 (0.44)	1.28 (0.44)	<0.0001	1.22 (0.45)	1.22 (0.45)	1.23 (0.45)	<0.0001	1.3 (0.43)	1.27 (0.42)	1.33 (0.44)	<0.0001
HbA1c (%)	5.10 (0.90)	5.10 (0.80)	5.00 (0.80)	<0.0001	5.00 (0.80)	5.10 (0.90)	5.00 (0.90)	<0.0001	5.10 (0.90)	5.20 (0.90)	5.00 (0.80)	<0.0001

Abbreviations: SBP—systolic blood pressure, DBP—diastolic blood pressure, FPG—fasting plasma glucose, TC—total cholesterol, TG—triglyceride, LDL-C—low-density lipoprotein cholesterol, HDL-C—high-density lipoprotein cholesterol, HbA1c—glycosylated hemoglobin.

**Table 3:** Adiposity indexes of participants in CNHS 2015–2017.

Characteristics	Overall				Male				Female			
	Total	Hypertension	No Hypertension	*p*-Value	Total	Hypertension	No Hypertension	*p*-Value	Total	Hypertension	No Hypertension	*p*-Value
CVAI	98.68 (55.56)	110.64 (52.93)	85.78 (51.20)	<0.0001	93.84 (65.39)	105.41 (63.21)	81.51 (61.74)	<0.0001	101.80 (48.65)	113.90 (45.29)	88.51 (43.76)	<0.0001
VAI	1.56 (1.73)	1.74 (1.94)	1.39 (1.48)	<0.0001	1.25 (1.42)	1.36 (1.57)	1.13 (1.25)	<0.0001	1.86 (1.90)	2.13 (2.15)	1.62 (1.60)	<0.0001
LAP	27.65 (34.39)	33.44 (39.31)	22.35 (27.84)	<0.0001	23.12 (32.41)	27.90 (36.60)	18.68 (26.52)	<0.0001	31.55 (35.13)	38.18 (40.03)	25.50 (27.91)	<0.0001
ABSI (m^11/6^/kg^2/3^)	0.0788 (0.0065)	0.0793 (0.0064)	0.0781 (0.0065)	<0.0001	0.0790 (0.0062)	0.0795 (0.0061)	0.0784 (0.0062)	<0.0001	0.0786 (0.0068)	0.0792 (0.0068)	0.0779 (0.0067)	<0.0001
BRI	3.83 (1.64)	4.13 (1.67)	3.51 (1.50)	<0.0001	3.60 (1.55)	3.89 (1.56)	3.32 (1.43)	<0.0001	4.03 (1.70)	4.36 (1.73)	3.69 (1.54)	<0.0001
CI (m^2/3^/kg^1/2^)	1.23 (0.12)	1.25 (0.11)	1.21 (0.11)	<0.0001	1.23 (0.11)	1.25 (0.11)	1.22 (0.11)	<0.0001	1.23 (0.12)	1.25 (0.11)	1.21 (0.11)	<0.0001
WC (cm)	83.4 (13.55)	85.70 (13.50)	81.00 (13.00)	<0.0001	84.65 (14.15)	87.00 (13.92)	82.20 (13.55)	<0.0001	82.4 (13.15)	84.85 (13.00)	80.05 (12.25)	<0.0001
BMI (kg/m2)	24.2 (4.68)	24.97 (4.80)	23.43 (4.31)	<0.0001	23.95 (4.61)	24.71 (4.67)	23.21 (4.26)	<0.0001	24.41 (4.76)	25.21 (4.88)	23.63 (4.35)	<0.0001

Abbreviations: CVAI—Chinese visceral adiposity index, VAI—visceral adiposity index, LAP—lipid accumulation product index, ABSI—a body shape index, BRI—body roundness index, CI—conicity index, WC—waist circumference, BMI—body mass index.

**Table 4:** Associations between the quartile (Q) of adiposity indexes and hypertension in CHNS 2015–2017.

Indicators	Group of Quartile	N	No. of Cases	Hypertension OR (95% CI)		
				Model 1	Model 2	Model 3
CVAI	Q1	24,800	8179	reference	reference	reference
	Q2	24,800	11,338	1.71 (1.65, 1.78)	1.60 (1.54, 1.66)	1.55 (1.50, 1.61)
	Q3	24,801	14,268	2.75 (2.65, 2.86)	2.43 (2.34, 2.52)	2.30 (2.21, 2.39)
	Q4	24,800	17,771	5.14 (4.95, 5.34)	4.12 (3.96, 4.29)	3.70 (3.54, 3.86)
	*p*-trend	-	-	<0.0001	<0.0001	<0.0001
	Per 1SD	-	-	1.87 (1.84, 1.9)	1.74 (1.72, 1.77)	1.68 (1.66, 1.71)
VAI	Q1	24,800	10,630	reference	reference	reference
	Q2	24,800	12,115	1.27 (1.23, 1.32)	1.27 (1.23, 1.32)	1.24 (1.19, 1.28)
	Q3	24,801	13,561	1.61 (1.55, 1.67)	1.65 (1.59, 1.72)	1.53 (1.47, 1.59)
	Q4	24,800	15,250	2.13 (2.05, 2.21)	2.31 (2.22, 2.39)	1.95 (1.87, 2.05)
	*p*-trend	-	-	<0.0001	<0.0001	<0.0001
	Per 1SD	-	-	1.27 (1.25, 1.29)	1.31 (1.29, 1.33)	1.18 (1.16, 1.2)
LAP	Q1	24,798	9354	reference	reference	reference
	Q2	24,802	11,827	1.51 (1.45, 1.56)	1.59 (1.53, 1.65)	1.56 (1.50, 1.62)
	Q3	24,801	13,971	2.13 (2.06, 2.21)	2.27 (2.19, 2.35)	2.18 (2.09, 2.27)
	Q4	24,800	16,404	3.23 (3.11, 3.35)	3.67 (3.53, 3.81)	3.42 (3.27, 3.58)
	*p*-trend	-	-	<0.0001	<0.0001	<0.0001
	Per 1SD	-	-	1.53 (1.51, 1.56)	1.62 (1.59, 1.64)	1.54 (1.51, 1.57)
ABSI	Q1	24,799	10,875	reference	reference	reference
	Q2	24,801	12,358	1.27 (1.23, 1.32)	1.22 (1.18, 1.27)	1.17 (1.13, 1.21)
	Q3	24,801	13,765	1.60 (1.54, 1.66)	1.47 (1.42, 1.53)	1.36 (1.31, 1.41)
	Q4	24,800	14,558	1.82 (1.76, 1.89)	1.47 (1.42, 1.53)	1.35 (1.3, 1.4)
	*p*-trend	-	-	<0.0001	<0.0001	<0.0001
	Per 1SD	-	-	1.25 (1.23, 1.26)	1.14 (1.13, 1.16)	1.11 (1.1, 1.13)
BRI	Q1	24,800	9045	reference	reference	reference
	Q2	24,805	11,496	1.51 (1.45, 1.56)	1.57 (1.52, 1.63)	1.49 (1.44, 1.55)
	Q3	24,801	14,130	2.31 (2.23, 2.39)	2.38 (2.29, 2.47)	2.16 (2.08, 2.25)
	Q4	24,795	16,885	3.72 (3.58, 3.86)	3.68 (3.54, 3.83)	3.18 (3.06, 3.31)
	*p*-trend	-	-	<0.0001	<0.0001	<0.0001
	Per 1SD	-	-	1.68 (1.66, 1.71)	1.70 (1.68, 1.73)	1.61 (1.59, 1.63)
CI	Q1	24,800	9877	reference	reference	reference
	Q2	24,800	12,134	1.45 (1.40, 1.5)	1.45 (1.40, 1.51)	1.36 (1.31, 1.41)
	Q3	24,801	13,867	1.92 (1.85, 1.99)	1.87 (1.80, 1.94)	1.68 (1.62, 1.75)
	Q4	24,800	15,678	2.60 (2.50, 2.69)	2.28 (2.2, 2.37)	1.99 (1.91, 2.06)
	*p*-trend	-	-	<0.0001	<0.0001	<0.0001
	Per 1SD	-	-	1.43 (1.41, 1.45)	1.36 (1.34, 1.38)	1.29 (1.27, 1.31)
WC	Q1	24,868	9518	reference	reference	reference
	Q2	24,793	11,549	1.41 (1.36, 1.46)	1.55 (1.49, 1.61)	1.48 (1.42, 1.54)
	Q3	24,785	13,819	2.03 (1.96, 2.11)	2.26 (2.18, 2.35)	2.07 (1.99, 2.15)
	Q4	24,755	16,670	3.33 (3.20, 3.45)	3.73 (3.59, 3.88)	3.25 (3.12, 3.38)
	*p*-trend	-	-	<0.0001	<0.0001	<0.0001
	Per 1SD	-	-	1.59 (1.57, 1.61)	1.68 (1.66, 1.7)	1.60 (1.57, 1.62)
BMI	Q1	24,800	9815	reference	reference	reference
	Q2	24,802	11,475	1.32 (1.27, 1.36)	1.51 (1.45, 1.57)	1.45 (1.39, 1.5)
	Q3	24,799	13,636	1.87 (1.80, 1.93)	2.27 (2.19, 2.36)	2.10 (2.02, 2.18)
	Q4	24,800	16,630	3.11 (3.00, 3.22)	4.02 (3.87, 4.18)	3.55 (3.41, 3.70)
	*p*-trend	-	-	<0.0001	<0.0001	<0.0001
	Per 1SD	-	-	1.57 (1.55, 1.59)	1.75 (1.72, 1.77)	1.51 (1.49, 1.54)

Model 1: unadjusted model; Model 2: adjusted for age and gender; Model 3: further adjusted for geographic region (urban or rural), education, marital status, income, family history, smoking, alcohol drinking, sleeping time, physical activity, sedentary behavior, medical examination within one year, diabetes, and dyslipidemia.

**Table 5:** Comparison of AUCs for anthropometric indexes in predicting hypertension in males and females.

	Male						Female					
	AUC	95%CI	Cut-Off Point	Sensitivity	Specificity	Youden	AUC	95%CI	Cut-Off Point	Sensitivity	Specificity	Youden
CVAI	0.636	0.631–0.641	98.268	0.562	0.638	0.199	0.706	0.702–0.710	101.165	0.653	0.651	0.304
VAI	0.561 *	0.556–0.566	1.358	0.503	0.592	0.095	0.602 *	0.597–0.607	1.937	0.552	0.599	0.152
LAP	0.610 *	0.605–0.615	21.599	0.604	0.558	0.162	0.641 *	0.637–0.646	30.272	0.621	0.588	0.209
ABSI	0.564 *	0.559–0.569	0.078	0.644	0.455	0.099	0.570 *	0.565–0.575	0.079	0.551	0.558	0.109
BRI	0.633 *	0.628–0.638	3.608	0.595	0.605	0.200	0.651 *	0.647–0.656	4.099	0.586	0.638	0.224
CI	0.598 *	0.593–0.604	1.225	0.610	0.539	0.149	0.608 *	0.603–0.61	1.223	0.608	0.552	0.160
WC	0.622 *	0.617–0.627	84.450	0.595	0.587	0.181	0.634 *	0.630–0.639	82.875	0.582	0.621	0.203
BMI	0.620 *	0.615–0.625	24.096	0.571	0.610	0.181	0.625 *	0.620–0.630	24.516	0.577	0.608	0.186

* Compared with the AUC of CVAI, *p* < 0.01.

## Data Availability

According to the policy of the National Institute for Nutrition and Health, China CDC, data related to this research are not allowed to be disclosed.

## Supplementary Materials

The following supporting information can be downloaded at: https://www.mdpi.com/article/10.3390/nu15092146/s1, Figure S1: Dose–response relationships between VAI, LAP, ABSI, BRI, CI, WC, BMI, and risk of hypertension by restricted cubic spline in Chinese 45 years and older males (**A**) and females (**B**).
