# Supplementary material for: Comparison of Several Adiposity Indexes in Predicting Hypertension among Chinese Adults: Data from China Nutrition and Health Surveillance (2015–2017)
Source: Nutrients. 2023 Apr 29;15(9):2146. doi: 10.3390/nu15092146 (PMC10180534; doi:10.3390/nu15092146)
Supplement: Supplementary file 1 [file nutrients-15-02146-s001.zip › nutrients-2361610-supplementary.pdf]

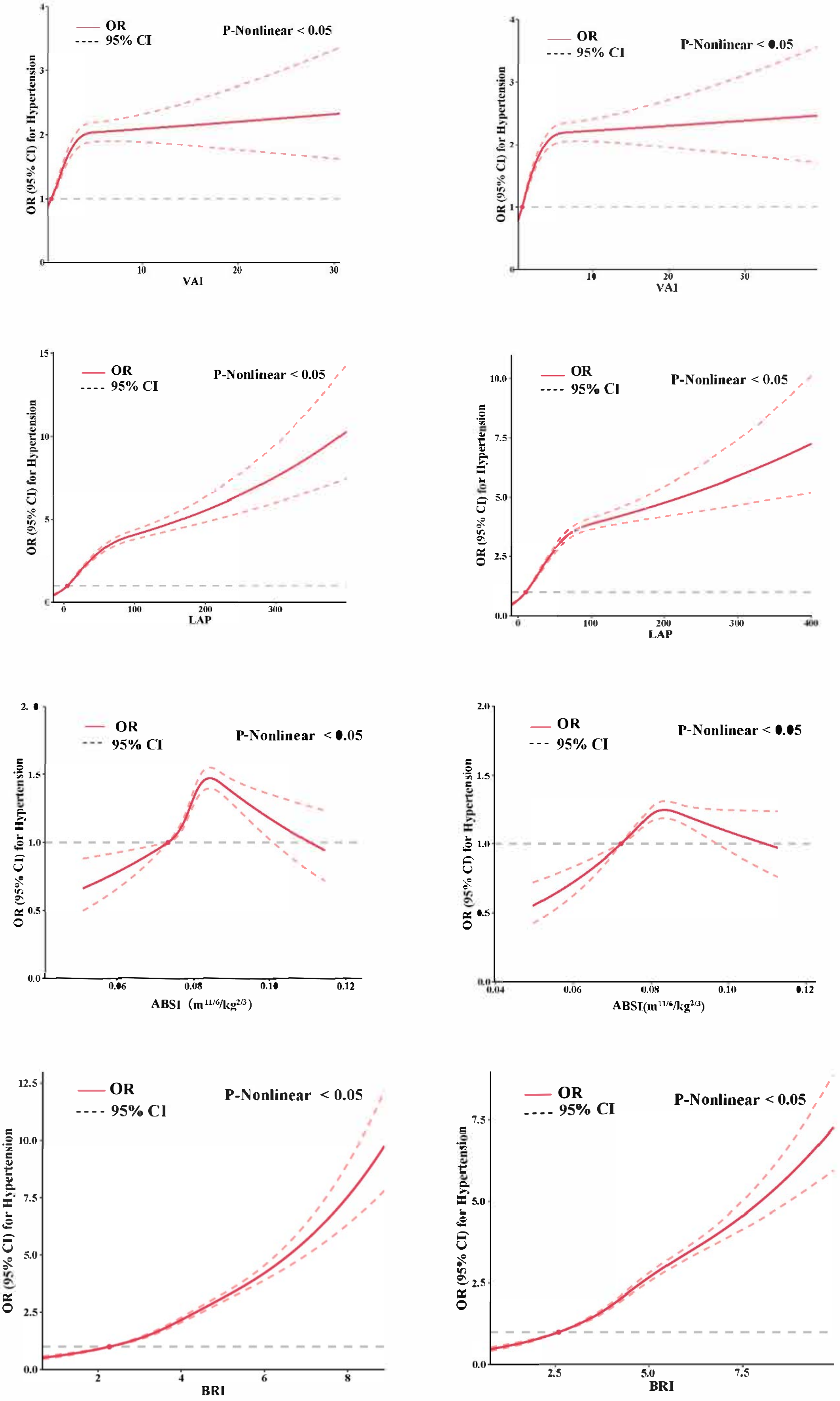

OR
95% CI
P-Nonlinear < 0.05
OR (95% CI) for Hypertension
VAI
OR
95% CI
P-Nonlinear < 0.05
OR (95% CI) for Hypertension
VAI
OR
95% CI
P-Nonlinear < 0.05
OR (95% CI) for Hypertension
LAP
OR
95% CI
P-Nonlinear < 0.05
OR (95% CI) for Hypertension
LAP
OR
95% CI
P-Nonlinear < 0.05
OR (95% CI) for Hypertension
ABSI (m11/6/kg2/3)
OR
95% CI
P-Nonlinear < 0.05
OR (95% CI) for Hypertension
ABSI(m11/6/kg2/3)
OR
95% CI
P-Nonlinear < 0.05
OR (95% CI) for Hypertension
BRI
OR
95% CI
P-Nonlinear < 0.05
OR (95% CI) for Hypertension
BRI

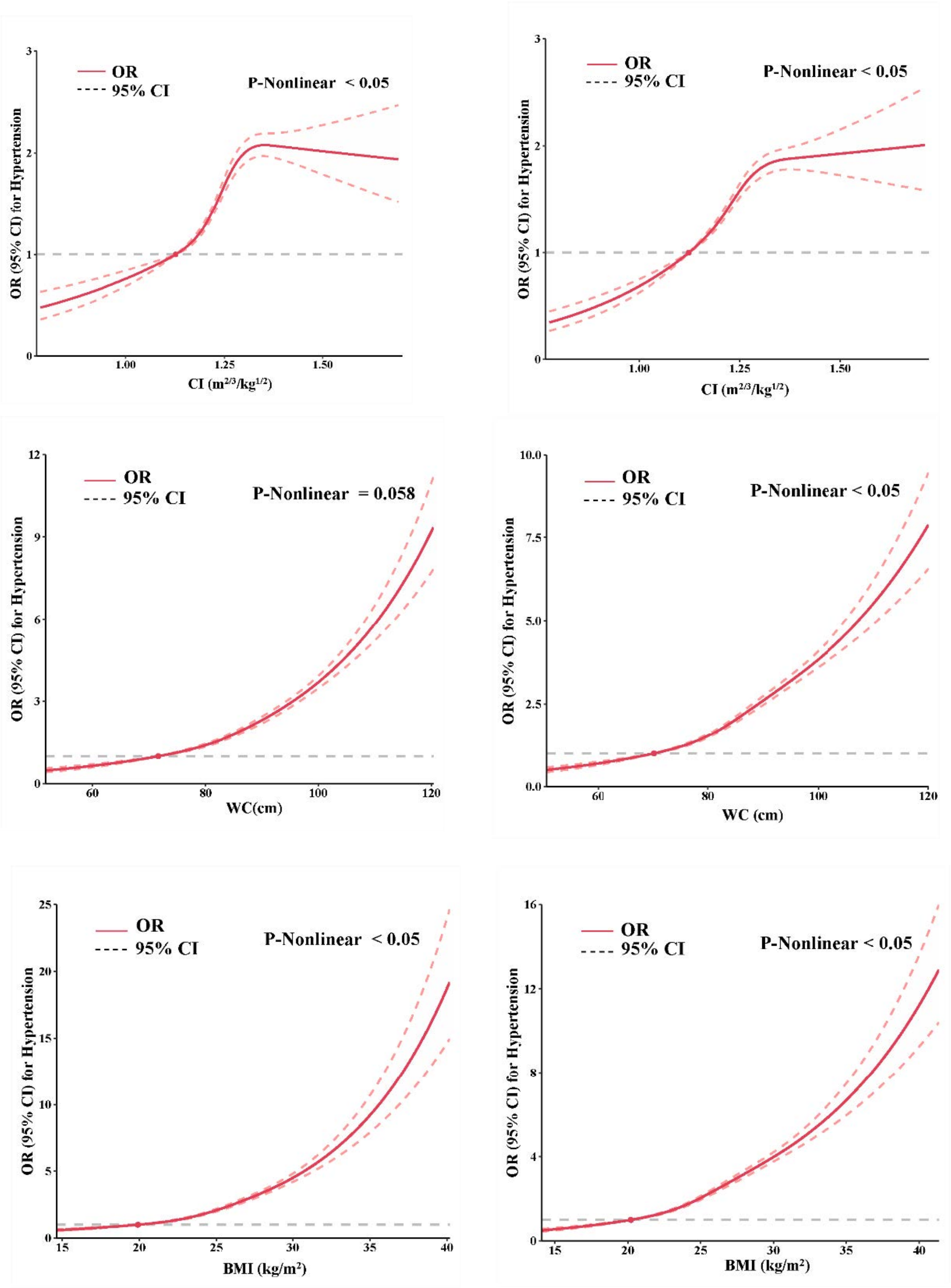

(**A**) (**B**)

Supplemental Figure S1 Dose–response relationships between VAI, LAP, ABSI, BRI, CI, WC, and BMI and risk of hypertension by restricted cubic spline in Chinese 45-years and older males **(A)** and females **(B)**. The associations were adjusted for age, geographic region, education, marital status, income, family history, smoking, alcohol-drinking, sleeping time, physical activity, sedentary behavior, medical examination within one year, diabetes and dyslipidemia. The red solid lines and red dashed lines represent the estimated ORs and their 95% CIs respectively. The gray dashed lines represent the corresponding adiposity indexes when the OR is 1.
